# In Vitro Destruction of Pathogenic Bacterial Biofilms by Bactericidal Metallic Nanoparticles via Laser-Induced Forward Transfer

**DOI:** 10.3390/nano10112259

**Published:** 2020-11-15

**Authors:** Alena Nastulyavichus, Eteri Tolordava, Andrey Rudenko, Darya Zazymkina, Pavel Shakhov, Nikolay Busleev, Yulia Romanova, Andrey Ionin, Sergey Kudryashov

**Affiliations:** 1 Laboratory of Laser Nanophysics and Biomedicine, P.N. Lebedev Physical Institute of the Russian Academy of Sciences, 119991 Moscow, Russia; tolordava-eteri@yandex.ru (E.T.); aa_rudenko@mail.ru (A.R.); zazymkina_darya@mail.ru (D.Z.); pashamyl999@gmail.com (P.S.); busleevni@lebedev.ru (N.B.); ioninaa@lebedev.ru (A.I.); kudryashovsi@lebedev.ru (S.K.); 2 Laboratory of Genetic Engineering of Pathogenic Microorganisms, N.F. Gamaleya Federal Research Centre of Epidemiology and Microbiology, 123098 Moscow, Russia; genes2007@yandex.ru

**Keywords:** laser-induced forward transfer, metal nanoparticles, *Staphylococcus aureus* and *Pseudomonas Aeruginosa* bacterial biofilm, bactericidal effect

## Abstract

A novel, successful method of bactericidal treatment of pathogenic bacterial biofilms in vitro by laser-induced forward transfer of metallic nanoparticles from a polyethylene terephthalate polymeric substrate was suggested. Transferred nanoparticles were characterized by scanning and transmission electron microscopy, energy-dispersive X-ray and Raman spectroscopy. The antibacterial modality of the method was tested on Gram-positive (*Staphylococcus aureus*) and Gram-negative (*Pseudomonas Aeruginosa*) bacterial biofilms in vitro, revealing their complete destruction. The proposed simple, cost-effective and potentially mobile biofilm treatment method demonstrated its high and broad bactericidal efficiency.

## 1. Introduction

Antibiotics, being the most widely used drugs in the fight against pathogenic microorganisms, are eventually losing their activity [1,2], resulting in chronic infections, serious health problems and fatal diseases. Such resistance of pathogenic microorganisms to antibiotics is related to self-organization into structurally complex communities termed biofilms on diverse surfaces in natural, medical, and industrial settings. Biofilms cells are encased in a protective extracellular polysaccharide matrix produced by the bacteria themselves. The biofilm formation process consists of different stages: adherence/adhesion/attachment, aggregation/maturation/accumulation, and detachment/dispersal phase. The last step is the dispersal of mature biofilm-embedded bacteria out of the biofilm [3], infecting medical devices such as catheters or implants [4,5,6], human organs such as teeth, skin, and the urinary tract [6,7].

Bacterial biofilms, due to their complex structure and dangerous resistance to antibiotics, are in focus of the scientific community and require always novel approaches in their treatment [8,9]. To date, various methods were proposed to treat bacterial biofilms, or to prevent their appearance. Quite successful is the use of chemical and photodynamic treatment methods [10,11,12], hydrophobic and topographic pre-treatment of substrates [13,14,15], antibacterial metal and semiconductor nanoparticles (NPs) [16,17,18,19,20,21], which are also promising as metal-polymer nanoparticle-based composites (nanomaterials) in a variety of biomedical applications, including drug delivery, microfluidic valve control, and cancer therapy [22,23,24,25]. Metal and metal oxide nanoparticles represent a group of materials, which were investigated in respect to their antimicrobial effects [26]. Ag NPs are the most popular inorganic nanoparticles used as antimicrobial agents [27,28]. CuO nanoparticles also exhibited inhibitory effects against Gram-positive and Gram-negative bacteria [29,30]. Carbon-based nanoparticles were reported to exhibit high antimicrobial activity as well, causing membrane damage in bacteria due to an oxidative stress [31]. In addition, the nanoparticles can be accumulated on the bacterial surface occurs due to electrostatic force. The nanoparticles can bind and penetrate the negative charged bacterial cell membrane to enter cell [32]. Furthermore, polymers and their composites were actively studied as antimicrobial and biodegradable biomaterials, e.g., chitosan [33]. The proposed mechanism for its antimicrobial action is binding to the negatively charged bacterial cell wall, with consequent destabilization of the cell envelope and altered permeability, followed by attachment to DNA with inhibition of its replication [33,34,35]. Metal nanoparticles can additionally improve the mechanical properties of polymers, due to the intrinsic characteristics of nanosized metals such as large surface area and high modulus [36]. For example, silver nanoparticles coated onto polyurethane foams demonstrated antibacterial activity as water filters [37]. Kim and co-authors [38] demonstrate enhancement of Raman and photoluminescence of core-shell hybrid Ag/polymer nanoparticles, consisting of Ag (core) and polydiacetylene (shell) through the assistance of localized surface plasmon effect for the effective biosensor. Meanwhile, extensive studies are still devoted to increase efficiency of bactericidal treatments and expand their spectra of bactericidal activity, make them robust, cost-effective and mobile.

In this article, a new method for treating pathogenic bacterial biofilms is publicly described [39], which invokes direct laser-induced forward transfer (LIFT) [40,41,42] of metal-polymer nanoparticles from a donor polymer substrate in a scanning mode onto acceptor pre-formed Gram-positive or Gram-negative bacterial biofilms. Similar studies carried out for metallic films deposited on donor silica glass substrates, were performed for comparison. This method is widely used for additive micropatterning [43,44]. LIFT has shown the ability to direct write different metals for interconnects and mask repair and also simple dielectric materials such as metal oxides [44,45]. Addition it is known that this technique adequate for the production of biosensors, since it permits to deposit patterns of biomolecules with high spatial resolution [46].

## 2. Materials and Methods

Bacterial biofilms were prepared, using an overnight broth culture of bacteria diluted 1:100 in Luria–Bertani (LB) culture medium by Miller, AppliChem, Germany. The diluted culture was added to test tubes with glass plates–1-cm × 1 cm wide pieces of glass slides. Incubated in a thermostat at 37 °C for 24 h with shaking 10 times per minute, with a tilt angle of 4°. Gram-positive (*Staphylococcus aureus*) and Gram-negative (*Pseudomonas aeruginosa*) cultures were taken for this research.

**Figure 1:**
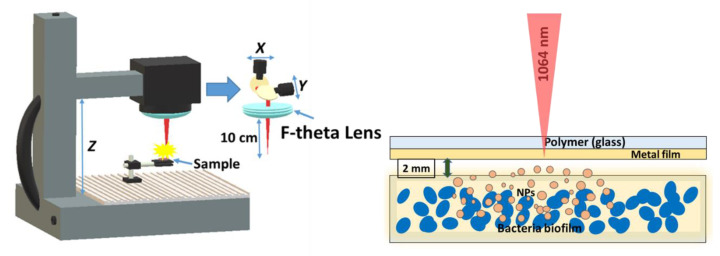
Experimental layout of laser-induced forward transfer of nanoparticles from a transparent substrate (polymer PET or silica glass) onto biofilms of pathogenic microorganisms.

During biofilm treatment, the glass plates with biofilms were fixed on glass slides and subjected to laser-induced forward transfer of silver, copper and gold metallic (Au, Ag, Cu) films as sputtered nanoparticles (Figure 1). 100-nm thick metallic films were obtained by magnetron sputtering of pure Au (99.99%), Ag (99.99%) and Cu (99.99%) targets in argon atmosphere onto 1-mm thick silica glass slides and 0.5-mm thick polymer (polyethylene terephthalate, PET) film substrates; film thicknesses were measured using a scanning probe microscope Certus Standard V (NanoScanTechnology). Air gap of ≈2 mm was established between the metal film and the glass substrate with a bacterial biofilm by means of a micro-positioning stage. 1064 nm nanosecond laser radiation (Yb^3+^–doped nanosecond fiber laser HTF MARK (Bulat), pulse width at half-height–120 ns) was focused by a f-theta objective lens (focal length–160 mm) into a spot with a 1/e-diameter ≈50 μm onto the metallic films on rear side of the polymer or silica glass substrates through these substrates, using a pulse energy of 0.2 mJ and repetition rate of 20 kHz, and scanned across the films at the speed of 1500 mm/s by a galvano-scanner (Figure 1). In each test, 1-cm × 1 cm wide spots of metallic films were transferred onto the biofilm in the scanning mode, in order to cover the entire front and rear surfaces of the biofilm sample. In addition to transferring nanoparticles onto the bacterial biofilms, another option was also considered, when the biofilm was grown on the silica glass substrates, pre-coated by silver, copper, or gold nanoparticles laser-transferred from the PET polymer substrate under the same conditions.

Surface topography and chemical composition of the transferred nanoparticles were characterized by their deposition onto a Si wafer surface, using a scanning electron microscope (SEM) JEOL 7001F, equipped by an energy-dispersion X-ray spectroscopy (EDX) module INCA (Oxford Instruments) for chemical micro-analysis. For transmission electron microscopy (TEM) studies, nanoparticles were deposited on a carbon mesh (TEM, JEOL JEM-2100F). Raman analysis was performed in range of 300–2100 cm^−1^, using a confocal Raman microscope Confotec MR 350 at the excitation wavelength of 532 nm.

The Live/Dead visualization tests on the treated bacterial biofilms were performed, using their subsequent coloration by “Live/Dead Biofilm Viability Kit” and a fluorescence microscope Nikon H600L with its 40× fluorescence objective lens (instrumental magnification–600×).

## 3. Results and Discussions

### 3.1. Nanoparticle Characterization

**Figure 2:**
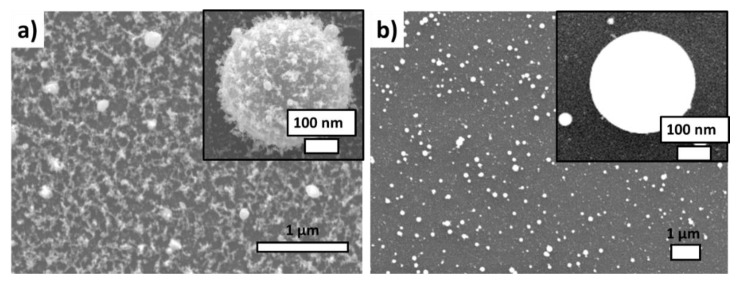
SEM visualization of Ag NPs laser-transferred onto the acceptor Si substrate: (**a**) from the donor polyethylene substrate, (**b**) from the donor silica glass substrate.

First, the metallic nanoparticles transferred by laser ablation of a metal film off the PET or glass substrates in air were characterized on Si wafer substrates by scanning and transmission electron microscopy. The spherical-shaped nanoparticles transferred from the PET substrate are characterized by the presence of “peculiar” fluffy caps on their surface (Figure 2a). In the case of the laser-induced forward transfer from the silica glass substrate, the metallic nanoparticles were also spherical, but at the same time very smooth (Figure 2b). The particle size in both cases varied in the range from 10 to 300 nm (the most probable size ≈ 100–150 nm); their almost perfect spherical shapes indicated the melting of their precursor ablation products from the island-like metallic films prior the transfer and their hydrodynamic relaxation during the transfer to the minimal surface energy.

**Figure 3:**
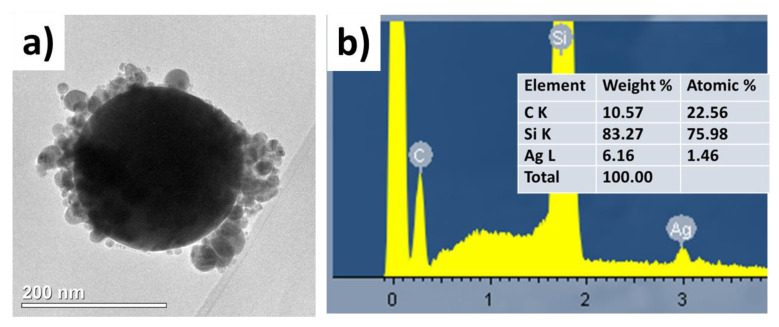
(**a**) TEM visualization of single Ag nanoparticle laser-transferred from the PET substrate; (**b**) Data (spectrum and datasheet) of its energy dispersive X-ray spectroscopy analysis on the acceptor Si substrate, which also appears in the spectrum.

Transmission electron microscopy has also revealed the presence of the carbonaceous shell on the surface of the metallic nanoparticles transferred from the polymer substrate (Figure 3a), while the accompanying energy dispersive x-ray analysis demonstrated the presence of the sufficiently large (about 23 atomic percent) content of carbon (Figure 3b). 

**Figure 4:**
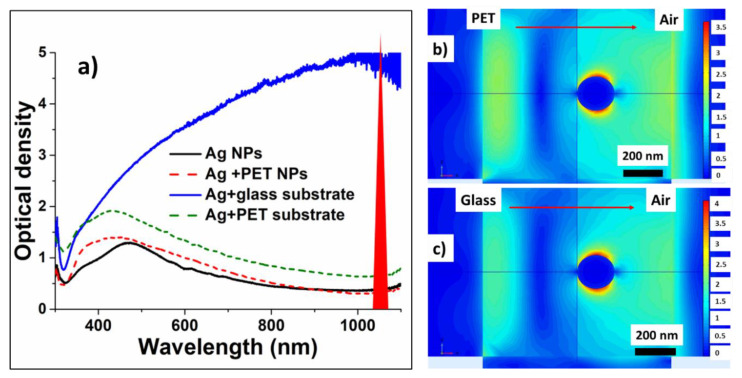
(**a**) Optical density spectra of Ag nanoislands on donor PET and glass substrates and of their corresponding deposits on acceptor glass slides; (**b**,**c**) Normalized field distributions around 200-nm sized Ag NPs, supported on PET and glass substrates, in the field of the 1064-nm plane wave. Right-hand color scales show the field enhancement magnitude.

The incident ns-laser intensities, required to lift-off Ag NPs from the PET and glass substrates, are rather low and very different- ~10 MW/cm^2^ and ~10^2^ MW/cm^2^, respectively. Simultaneously, the optical density of the initial precursor films at the pump laser wavelength of 1064 nm varies by a few time much higher for the glass-supported nanoparticles due to aggregation peculiarities on the glass surface) (Figure 4a) for the same film deposition conditions. Meanwhile, despite the laser wavelength is far from the spectral position of localized plasmon resonance for Ag NPs (≈400 nm), the resulting transferred and deposited nanoparticles exhibit in this figure the almost same optical density spectra in both these cases, indicating the same LIFT efficiency for the same initial film thicknesses and the same final state of nanoparticles in the deposits.

To provide insight into the underlying laser physics, in this work a numerical simulation based on a finite element method (FEM) was carried out to characterize the spatial electric field distribution. We used linearly polarized plane wave with the wavelength 1064 nm for illuminating an Ag nanosphere located on a semi-infinite dielectric substrate (either PET, or silica glass). The radius of the nanosphere was 100 nm and the vertical length of the computational cell was 1 μm. A perfectly matched layer (PML) boundary condition was applied for the computational domain. Our FEM simulations demonstrate considerable near-field enhancement of the incident plane 1064 nm wave in the plasmonic dipolar mode of the nanoparticles by 19 (glass) and 14 (PET) times for the field amplitude (Figure 4b,c). This yields in rather strong heating and thermal expansion of the 200-nm sized nanoparticles, driving their center-of-mass “hopping” lift-off [47]. Moreover, the observed carbonaceous debris indicates that ablation of the substrates (more strongly-PET one) apparently occurs in the contact region underneath the nanoparticles, driving another, “trampoline” NP-removal mechanism [47].

**Figure 5:**
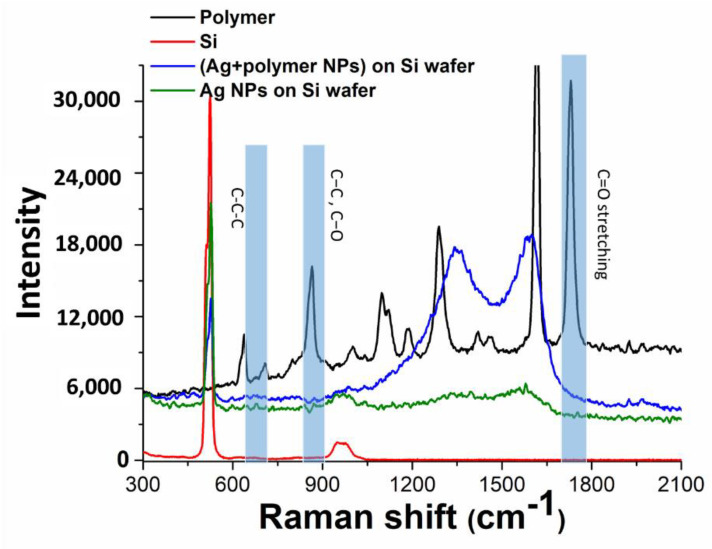
Raman spectra of samples: donor PET and acceptor Si substrates, Ag NPs transferred onto acceptor Si substrates from the donor PET and glass substrates.

Chemical composition of the carbonaceous residue around the Ag nanoparticles transferred onto monocrystalline silicon substrates either from the glass, or the polymer substrates was analyzed by Raman micro-spectroscopy in the range of 300–2100 cm^−1^ (Figure 5), using as a reference the Raman spectrum of PET with its main bands at 1625 cm^−1^ (C=C stretching) and 1736 cm^−1^ (carbonyl (C=O) stretching) (see also Table 1) [48,49].

Regarding Ag nanoparticles, in agreement with SEM and TEM visualization such nanoparticles transferred from the PET substrate, exhibit in Figure 5 much stronger carbonaceous contamination, comparing to nanoparticles transferred from the silica glass substrate. Specifically, both types of nanoparticle deposits give rise to two main bands in the spectra, which are typical for glassy carbon with the characteristic D-band at 1340 cm^−1^ (vibrations of carbon atoms with dangling bonds for the in-plane terminated disordered graphite) and G-band at 1593 cm^−1^ (E_2g_ mode of the two-dimensional hexagonal graphitic structure) [50], representing the coexistence of sp^2^ and sp^3^-coordineted carbon in the residue. The same, but weak Raman bands in the spectrum of the Ag nanoparticles, transferred from the carbon-free silica glass substrate, can be related to post-contamination of the sample during its storage and analysis.

### 3.2. Microbiological Tests

**Figure 6:**
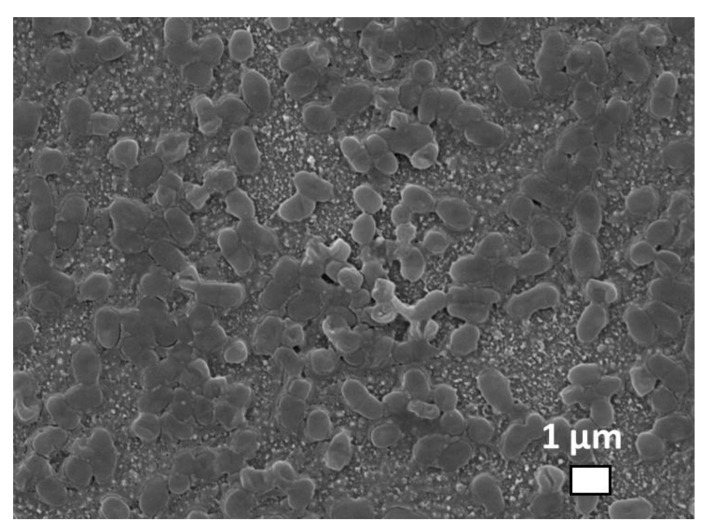
SEM visualization of *Staphylococcus aureus* bacteria incubated on the glass substrate with predeposited Ag nanoparticles.

These in vitro studies were performed on biofilms of Staphylococcus aureus and Pseudomonas aeruginosa bacteria. Figure 6 shows 1-micron sized separate Staphylococcus aureus bacteria incubated on a silica glass slide with pre-deposited silver nanoparticles, with an additional 10 nm gold layer deposited atop the sample to eliminate surface charging during the scanning electron microscope visualization.

During the LIFT-based biofilm treatment, the clean gold, silver and copper metallic nanoparticles from the silica glass substrates, as well as same particles coated with fluffy PET were laser-transferred to the previously grown daily biofilm samples. After the laser-induced nanoparticle transfer, the glass plates with the bacterial biofilms were transferred to test tubes with a physiological saline solution and the basics were shaken vigorously on a shaker for 1 h. Under the influence of DNA-ase, the biofilm matrix was destroyed, but the bacteria cells remain unharmed. Then, the resulting suspension was titrated by the standard microbiological method, prepared ten-fold dilutions and sown on a solid nutrient medium to determine corresponding CFU (colony forming unit) values (Table 2). All samples with biofilms were grown in the same conditions. To determine the degree of antibacterial effect of nanoparticles on biofilms, experimental samples were compared with biofilms of untreated nanoparticles (control of biofilm growth). To evaluate the effect of the direct laser radiation on the biofilm, scanning of the transparent substrates was performed under similar conditions, but without the metallic films (Table 2, PET). According to the results in Table 2, direct laser irradiation didn’t influence the films.

With a well-expressed antibacterial effect, the number of bacteria decreases by several orders of magnitude. Studies have shown that silver and copper nanoparticles transferred from the polymer substrate completely destroyed the biofilms in both *Staphylococcus aureus* and *Pseudomonas aeruginosa*. The number of bacteria in these samples decreased by 6–7 orders of magnitude. In contrast, gold nanoparticles transferred from the PET substrate did not show a decrease in CFU for gram-positive and gram-negative bacteria. In the case of laser-induced direct transfer of metal nanoparticles from a quartz glass substrate, the result was almost similar to the control experiment, in which the biofilms were not exposed to laser radiation or nanoparticles. A slight decrease in the number of bacteria was observed when silver and copper were transferred from the quartz glass substrate. In these samples, the number decreased by only one order of magnitude and only in the case of Pseudomonas aeruginosa. 

**Figure 7:**
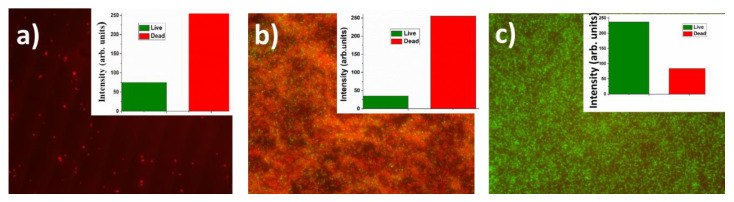
Optical images of assays of live (green) and dead (red) cells in Staphylococcus aureus bacterial biofilms incubated on silica glass slides pre-coated by metallic nanoparticles transferred from the PET substrate: (**a**) Ag; (**b**) Cu; (**c**) Au (the instrumental magnification–600×). Insets: RGB analysis of live and dead bacteria. Image size-30 × 25 μm.

Similarly to the case of metallic nanoparticle transfer onto the biofilms, very significant bactericidal effect (red staining) was noticeable for silver and copper nanoparticles, which coated glass slides for bacterial incubation (Figure 7), while for gold nanoparticles no effect was observed. However, comparing to the completely successful frontal laser-induced Ag and Cu nanoparticle transfer onto the biofilms, in the latter procedure the biofilm formation was not totally prevented, just delayed until a few layers of dead bacteria will isolate the biotoxic Ag or Cu-nanoparticle coating.

Finally, one can draw a few important conclusions, based on these experimental results. First, laser radiation itself doesn’t affect the films, as shown in Table 2 (PET column). Second, chemically-inert gold nanoparticles make very little effect both in the cases of PET and silicas glass substrates. Meanwhile, third, in the bactericidal effect of silver and copper nanoparticles there is apparently strong effect of their fluffy PET coating, which is yet to be unveiled by IR and Raman micro-spectroscopy, similarly to our previous studies [51]. Possible mechanism of bacteria death is not clear at this moment, but maybe connected to the property of metals to participate in redox reactions determining the tendency to acquire electrons from a donor [34]. Redox-active essential metals, such as silver and copper, can therefore act as catalytic cofactors in a wide range of cell enzymes either generating or catalyzing reactive oxygen species. Reactive oxygen species can induce an oxidative stress, damaging cellular proteins, lipids and DNA, if the cell antioxidant capacity is exceeded. Moreover, diffusion of metallic ions can cause structural changes and finally, bacterial death [34].

Importantly, this LIFT-based approach demonstrates very promising anti-fouling efficiency and biosafely, comparing to other well-known and patented laser-based nanotwechnological approaches, e.g., application of bactericidal colloidal nanoparticles from their solutions [27,52], in the surface density of nanoparticles resided on the biofilm. Moreover, our approach does not show direct photolithitic laser influence on the biofilms, comparing to laser heating of deposited optically-absorbing, but chemically-inert plasmonic nanoparticles [53,54,55] or direct laser-driven shock-wave impinging of bactericidal nanopartciles into biofilms [56]. Furthermore, we believe that with mobile laser scanners the LIFT-based anti-fouling treatment can go mobile too.

## 4. Conclusions

This work presents a new, promising, simple, robust, cost-effective and highly efficient treatment method for *Staphylococcus aureus* and *Pseudomonas aeruginosa* bacterial biofilms. This method consists in laser-induced forward transfer of metal-polymer composite nanoparticles from thin silver, copper and gold metallic film on polyethylene terephthalate substrate (not on silica glass one) directly onto the biofilms. Microbiological tests revealed the complete destruction of these bacterial biofilms by the silver and copper nanoparticles, not by chemically inert gold nanoparticles. After the laboratory in vitro investigation of this approach on stand-alone pathogenic biofilms and its patent application, in continuation of this research we will study this approach on mouse wounds in vivo regarding its efficiency to chronic infections and biotoxicity.

## Figures and Tables

**Table 1:** PET Raman bands and their assignment.

Raman (cm^−1^)	Assignment
639	C–C–C in plane bending
870	C–C stretching (ring breathing), C–O stretching
1115	CH in plane bending (ring), C–O stretching
1290	C–C stretching (ring), C–O stretching
1625	C=C stretching (ring)
1736	C=O stretching

**Table 2:** Effect of transferred NPs on CFU/mL values of clinical isolates biofilms.

	Ag/PET	Cu/PET	Au/PET	PET	Ag/Glass	Cu/Glass	Au/Glass	Control
*S. aureus*	0	0	2 × 10^6^	4 × 10^6^	4 × 10^6^	4 × 10^6^	2 × 10^6^	4 × 10^6^
*P. aeruginosa*	0	0	3 × 10^7^	1 × 10^7^	4 × 10^6^	4 × 10^6^	3 × 10^7^	3 × 10^7^
